# Surgery

**Published:** 1866-03

**Authors:** 


### SURGERY.

*Endoscopy.*—Certainly one of the most ingenious applications of the art of direct exploration and physical examination of the condition of the blind passages and internal organs of the human body, which modern professional zeal and enterprise has discovered, is that known by the name of Endoscopy. It boldly attempts and succeeds in accomplishing the direct, ocular examination of narrow canals and obscure cavities which until now have never admitted during life the light of day, and which would seem to most persons, *a priori*, to be entirely beyond the study of the most inquisitive research applied in this way. But modern medical exploration has not yet reached its limit, it would seem, and by the method of which we are speaking it now reveals the secret lacunæ of the urethra, and even the interior of the bladder itself, to the most thorough and exact ocular scrutiny. As yet the practice of this method may be said to be in its infancy merely; it is in the hands of but few practitioners, but the results already attained are such as to warrant the hope that it will furnish the means of rational and successful treatment in some most serious complaints, which have hitherto been to a very great extent treated blindly and rudely.

Endoscopy is an art rather than a science, inasmuch as it consists merely in a method of exposing, by means of ingenious mechanism, to visual inspection and study, hidden regions hitherto unexplored in this way. To the late lamented Dr. John D. Fisher, of this city, belongs the credit of having first devised an apparatus for this purpose, identical in principle and similar in structure to that which is employed with so much success in France at the present time by Desormeaux, and by Dr. Cruise in Dublin. So long ago as 1824, Dr. Fisher contrived his instrument, but it does not seem in his hands to have attracted the attention it deserved, or to have accomplished the results of which it is plainly capable. At the present time, endoscopy is in active employment by both of the gentlemen who have taken it up since the decease of Dr. Fisher. Dr. Cruise's instrument is that which has produced the results with which we are best acquainted. and its structure is quite simple and easily understood.

The apparatus consists of a lantern for the purpose of illumination, and a system of tubes and instruments by which its light is made available. Dr. Cruise employs the flat flame of a petroleum lamp, placed with the edge towards the exploring tube. This lamp is enclosed in a wooden case, to avoid the inconvenience of the heat and the diffused light, with an opening opposite the flame on one side. In this opening a strong condensing lens is set, through which the light passes into the eye tube. This is a short tube, something like the tube of a microscope, but shorter, which is screwed to the side of the lantern opposite the opening. It is so jointed to the lantern, to which it is attached by its side, that it can be freely rotated, but in use it is generally kept in a horizontal position. The condensed light from the lantern, having entered the eye tube through the lateral opening, falls on a perforated mirror set in this tube at an angle of 45°, by which the ray is reflected and thrown away from the eye of the observer along the axis of the tube. A small central opening in the mirror enables the eye of the observer placed behind to follow the light to the deepest recesses to which it may be directed. For examination of the urethra, a tube, the narrow portion of which is of the size of a large catheter, and six inches long, is fitted to the end of the eye-tube. Just before junction with the eye-tube it gradually expands, so as to give it a diameter large enough for the practical application of the instrument. When this tube is adjusted in the manner described, and introduced into the urethra, the effect is most marvelous. The whole canal can be examined most carefully, and the slightest local affection of whatever kind can be made out with perfect distinctness. In his interesting account of this instrument and its uses, Dr. Cruise gives colored plates showing the appearances of this canal, both diseased and healthy, even as far back as the prostatic portion. Having obtained a fair view of any morbid condition of the urethra, Dr. Cruise proceeds to apply the requisite treatment directly to the part affected. This is done by means of an opening on one side of the dilated portion of the urethral tube, by which he is able to introduce instruments for the purpose as far as may be necessary. He thus avoids the necessity of applying remedies in the usual way, by which the whole urethra is subjected to the stimulus of an injection, or exposed to the danger of laceration in a case of narrow and obstinate stricture. In chronic blennorrhagia, for instance, he applies his caustic directly to the granular surface of the membranous portion of the urethra with as much accuracy as the oculist does to the granulations of an old conjunctivitis. He is able also to watch the effect of his treatment from day to day, and is thus enabled to graduate the strength of his applications to the actual condition of the affected part, instead of trusting to the uncertain guide of the sensations or statements of the patient. Surely this is a great advance in practical surgery!

Another class of affections which the endoscope has furnished the means for successfully treating is stricture of the urethra. The most obstinate resistance on the part of these sometimes impervious obstructions of this canal have yielded to the insinuating power of a fine bougie passed under the eye of the observer. Dr. Cruise mentions several instances of this. In one, M. Civiale had tried for twenty-eight days to pass a sound through the stricture without success. At the second attempt, M. Desormeaux, by the aid of the endoscope, passed a fine bougie through the constriction, and from that time the case went on favorably. In another case, of a patient 73 years old, repeated attempts by Dr. Cruise to pass a bougie failed, until he employed the endoscope, by which his efforts were finally crowned with success, and gradual dilatation was employed until the canal was enlarged to its full dimensions. By means of this instrument, also, internal urethrotomy is disarmed of most of its dangers, the knife being applied directly to the part requiring division under the direct observation of the operator. This operation has been very successful in these cases in the hands of M. Desormeaux, aud it will undoubtedly often obviate the necessity of perineal section or of puncture of the bladder.

But the use of the endoscope is not limited to the canal which evacuates the bladder—it is made to explore that organ itself. By means of a catheter with a short curve, furnished with an opening on its convex surface, supplied with a glass window, the light from the lantern is thrown directly into the bladder. In this way Dr. Cruise has succeeded in perfectly examining the prostatic portion, the fundus and greater part of the posterior surface of this organ. In one instance of inflamed bladder, he made the condition of the lining membrane as visible to another observer as, to use his own words, "the conjunctiva of an inflamed eye." The *experimentum crucis* to which Dr. Cruise was subjected by his colleague, Dr. McDonnell, would seem to settle the question of the capabilities of the instrument for the purposes of accurate examination. This gentleman placed in the bladder of a dead body several articles, of the nature of which Dr. Cruise was not previously informed. By means of the endoscope he was enabled to make them out to be a Minie bullet, a brass screw with a milled head, and a mass of plaster of paris! Surely accuracy of diagnosis can go no farther.

At the present day, when specialties are occupying the attention of so many industrious members of the medical profession, the endoscope promises to be fruitful of valuable results. Its use must require a certain amount of patience and dexterity which can hardly be looked for from every general practitioner. With the laryngoscope it cannot fail to reward the labors of those who, by a natural gift or by a special cultivation of the capabilities of the instrument, will be enabled to substitute positive knowledge for conjecture, and direct application of remedies for blind treatment, in the management of a class of cases, many of which are among the most serious which the surgeon is called upon to treat.—*Boston Med. and Surg. Jour.* December, 1865.

